# Evaluation of the Effectiveness of Digital Technology Interventions to Reduce Loneliness in Older Adults: Systematic Review and Meta-analysis

**DOI:** 10.2196/24712

**Published:** 2021-06-04

**Authors:** Syed Ghulam Sarwar Shah, David Nogueras, Hugo Cornelis van Woerden, Vasiliki Kiparoglou

**Affiliations:** 1 NIHR Oxford Biomedical Research Centre Oxford University Hospitals NHS Foundation Trust Oxford United Kingdom; 2 Radcliffe Department of Medicine University of Oxford Oxford United Kingdom; 3 EvZein Limited, Holley Crescent, Headington Oxford United Kingdom; 4 Public Health Agency Northern Ireland Belfast United Kingdom; 5 Division of Rural Health and Wellbeing University of the Highlands and Islands Inverness United Kingdom; 6 Institute of Nursing and Health Research Ulster University Belfast United Kingdom; 7 Nuffield Department of Primary Care Health Sciences University of Oxford Oxford United Kingdom

**Keywords:** loneliness, older people, digital technology, effectiveness, efficacy, evidence, systematic review, meta-analysis

## Abstract

**Background:**

Loneliness is a serious public health issue, and its burden is increasing in many countries. Loneliness affects social, physical, and mental health, and it is associated with multimorbidity and premature mortality. In addition to social interventions, a range of digital technology interventions (DTIs) are being used to tackle loneliness. However, there is limited evidence on the effectiveness of DTIs in reducing loneliness, especially in adults. The effectiveness of DTIs in reducing loneliness needs to be systematically assessed.

**Objective:**

The objective of this study is to assess the effectiveness of DTIs in reducing loneliness in older adults.

**Methods:**

We conducted electronic searches in PubMed, MEDLINE, CINAHL, Embase, and Web of Science for empirical studies published in English from January 1, 2010, to July 31, 2019. The study selection criteria included interventional studies that used any type of DTIs to reduce loneliness in adults (aged ≥18 years) with a minimum intervention duration of 3 months and follow-up measurements at least 3 months after the intervention. Two researchers independently screened articles and extracted data using the PICO (participant, intervention, comparator, and outcome) framework. The primary outcome measure was loneliness. Loneliness scores in both the intervention and control groups at baseline and at follow-up at 3, 4, 6, and 12 months after the intervention were extracted. Data were analyzed via narrative synthesis and meta-analysis using RevMan (The Cochrane Collaboration) software.

**Results:**

A total of 6 studies were selected from 4939 screened articles. These studies included 1 before and after study and 5 clinical trials (4 randomized clinical trials and 1 quasi-experimental study). All of these studies enrolled a total of 646 participants (men: n=154, 23.8%; women: n=427, 66.1%; no gender information: n=65, 10.1%) with an average age of 73-78 years (SD 6-11). Five clinical trials were included in the meta-analysis, and by using the random effects model, standardized mean differences (SMDs) were calculated for each trial and pooled across studies at the 3-, 4-, and 6-month follow-ups. The overall effect estimates showed no statistically significant difference in the effectiveness of DTIs compared with that of usual care or non-DTIs at follow-up at 3 months (SMD 0.02; 95% CI −0.36 to 0.40; *P*=.92), 4 months (SMD −1.11; 95% CI −2.60 to 0.38; *P*=.14), and 6 months (SMD −0.11; 95% CI −0.54 to 0.32; *P*=.61). The quality of evidence was very low to moderate in these trials.

**Conclusions:**

Our meta-analysis shows no evidence supporting the effectiveness of DTIs in reducing loneliness in older adults. Future research may consider randomized controlled trials with larger sample sizes and longer durations for both the interventions and follow-ups.

**International Registered Report Identifier (IRRID):**

RR2-10.1136/bmjopen-2019-032455

## Introduction

### Background

Loneliness is a multifaceted public health problem [1]. The burden of loneliness is high in some countries [2-9], and it is increasing in many other countries [10]. Loneliness is expected to rise because of lockdowns, quarantine, self-isolation, and social distancing measures that are being enforced in several countries to tackle the COVID-19 pandemic [11,12]. Therefore, tackling loneliness is imperative, and digital technology could play a major role in addressing loneliness [13].

Loneliness refers to an individual’s subjective feelings of a perceived discrepancy between actual and desired social relationships [14,15]. Although loneliness affects people of all ages [15,16], older, younger, and vulnerable people are affected more by it [7,17,18]. Risk factors of loneliness include demographic characteristics, social factors, and physical environments [17-19]. Loneliness enhances the risk of poor physical and mental health [14,20-23], dementia [24], premature mortality, and all-cause mortality [21], particularly in older adults [23]. In addition, the implications of loneliness include the high costs of health and well-being (eg, between £6429.00 [US $8074.80] and £9616.00 [US $12,077.70] per person per year in the United Kingdom) [25] as well as lost work days and productivity (eg, costing up to £2.5 billion [US $3.14 billion] per annum for employers in the United Kingdom) [26]. Therefore, it is imperative to tackle loneliness.

Loneliness is being addressed through a range of social [27] and technological interventions [28]. The latter type of interventions includes numerous and diverse types of digital apps, web-based social networking tools, sensors, and robots [29]. Although these tools use digital technology, they are heterogeneous in many aspects, including the means they provide to socially connect; the purposes for which they are used; the ways and methods of their application; the frequency of their use; and their users, who differ from each other in many traits such as demographic, social, and economic characteristics, and some may have physical and mental limitations. Therefore, these digital technology tools need to be systematically evaluated for their effectiveness in tackling loneliness.

Several published reviews have reported that digital technology interventions (DTIs) are effective in reducing loneliness [30-34]. However, some of these studies are weak and have a high risk of bias [35], and other studies have used a few selected technological interventions and covered literature published over a short span, such as the 3-year period from January 2010 to January 2013 [31].

There is limited evidence on the effectiveness of DTIs for loneliness [36], and there are calls for further research [32,33] to assess and identify the latest DTIs that are effective in reducing loneliness [34,36]. In addition, evaluation of the latest evidence on the effectiveness of DTIs in reducing loneliness is imperative from the perspectives of patients and their families and other stakeholders such as health and social care providers and health insurers [37].

### Study Objectives

The primary objective of this study is to assess the effectiveness of DTIs in reducing loneliness in adults. The secondary objective is to identify DTIs that are used to reduce loneliness in adults.

### Review Questions

The main research question was “Are DTIs effective for reducing loneliness in adults?” The secondary question was “What DTIs are used for reducing loneliness in adults?”

### Outcome Measures

The main outcome measure was loneliness. We extracted data on loneliness measured at both the baseline (before the intervention) and follow-ups (at least 3 months after the intervention) for the intervention groups and control groups, if any.

## Methods

### Study Design, Conduct, and Reporting

We undertook a systematic review and meta-analysis as suggested in the Cochrane Methods for Systematic Reviews of Interventions [38]. We have reported the findings in accordance with the PRISMA (Preferred Reporting Items for Systematic Reviews and Meta-Analyses) guidelines [39].

### Protocol Registration and Publication

We registered this systematic review and meta-analysis with the PROSPERO database on June 10, 2019 (registration ID: CRD42019131524) [40], and we published our protocol [37] before undertaking this study.

### Patient and Public Involvement

A patient and public manager affiliated with our research center reviewed the study protocol and provided suggestions that were incorporated into the protocol. We had no access to any patient diagnosed with loneliness; therefore, we could not include any patients or members of the public in the design and conduct of the study. However, the findings of this study will be disseminated as an open access publication that will be freely available to patients and everyone else globally.

### Definition of DTI

We define the term DTI as an intervention that applies digital technology, that is, the technology, equipment, and apps that process information in the form of numeric codes, usually a binary code [41].

### Eligibility Criteria

We selected studies that met our predefined eligibility criteria [37]. Study designs included interventional studies (randomized and nonrandomized) that investigated the effects of DTIs on loneliness. We included a range of DTIs, that is, computers, computer tablets, iPads, internet, web-based videos, communication, chatting, social groups, meetings, conferences and messages, sensors, social robots, smart mobile phones, social media tools, and the World Wide Web. We set 3 months as the minimum intervention duration and follow-up period. The research participants were adults, both male and female, aged 18 years or more. We included different settings, that is, residential dwellings, including private residences and care or nursing homes or centers in any country. The studies were limited to journal articles in English published from January 1, 2010, to July 31, 2019.

### Information Sources and Keywords

We electronically searched PubMed, MEDLINE, CINAHL, Embase, and Web of Science and covered the publication period from January 1, 2010, to July 31, 2019. We used an a priori list of keywords prepared in our preliminary literature searches [37]. The keywords were of 2 categories: medical condition or problem (ie, *loneliness*, *lonely*, *isolation*, *aloneness*, disconnect*, *solitude*, singleness*, *lonesomeness*, *solitariness*, and *remoteness*) and intervention or technology (ie, *digital*, technolog*, sensor*, robot*, *internet*, *social media*, *phone*, *online*, iPad*, tablet*, computer*, *electronic*, *web*, *video*, and *videoconference*), as reported in our published protocol [37].

### Literature Searches

First, we searched the keywords in the *subject headings* such as MeSH major terms in PubMed or equivalent terms in other databases (for detailed search history, see Multimedia Appendix 1). Thereafter, we searched for keywords in the *title* and *abstract* fields in the selected databases using 3 Boolean operators: “OR,” “AND,” and “NOT.” In addition, we hand searched the reference lists of the shortlisted articles. We wrote emails to the authors of 2 studies requesting for full copies of their research articles [42,43], which were gratefully emailed to us. We contacted the authors of 2 further studies for missing or additional data [44,45]. We had a good response from the authors of both studies, and data were thankfully provided for 1 study only [45]. We sought support from an expert librarian at our library for running literature searches.

### Study Selection

**Figure 1:**
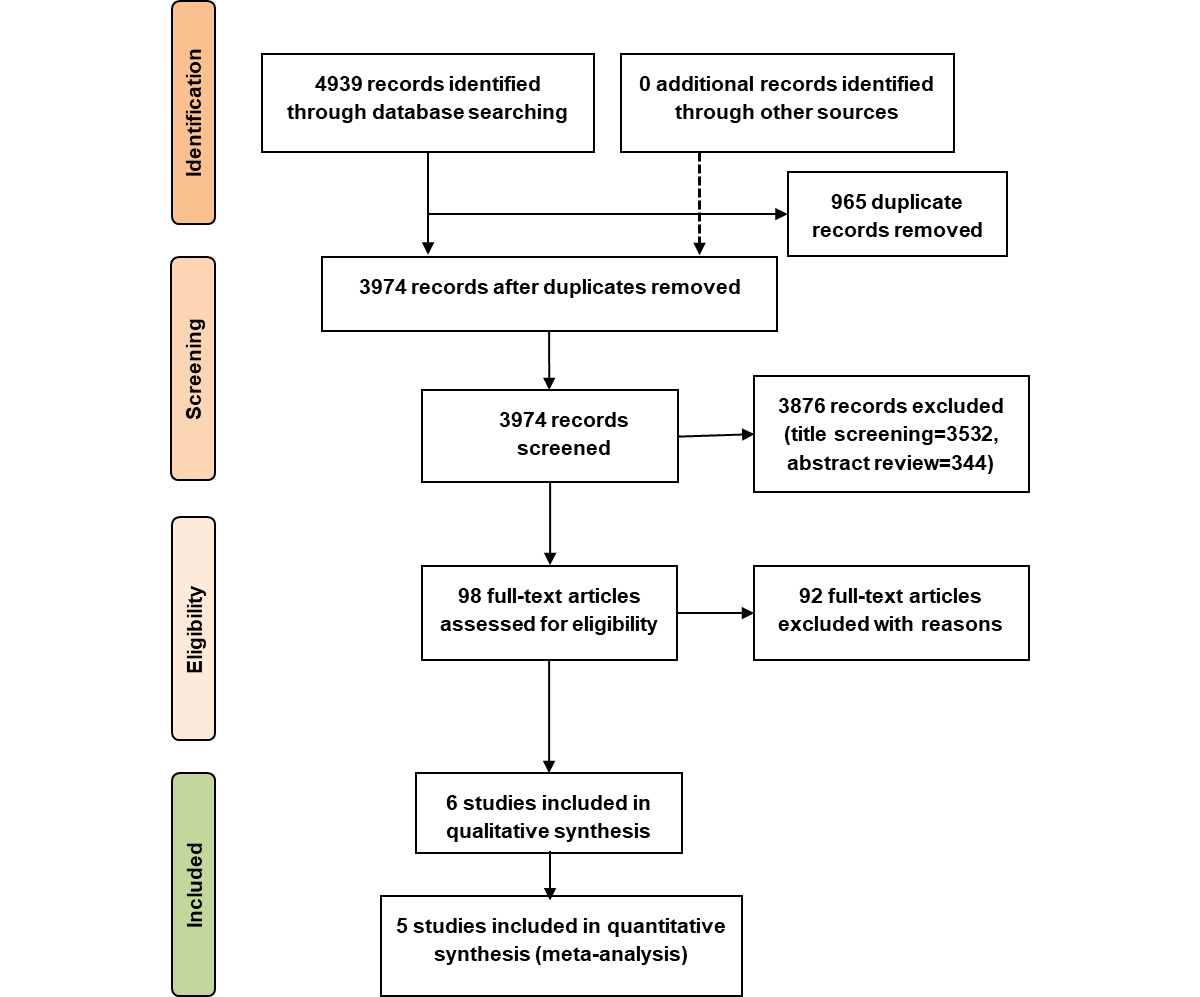
PRISMA (Preferred Reporting Items for Systematic Reviews and Meta-Analyses) study selection flow diagram.

Literature searches retrieved 4939 articles, of which 965 duplicate articles were removed (Figure 1). Two researchers (SGSS and DN) independently screened the remaining articles (n=3974) by title, which was followed by reading the abstracts of 442 articles (Figure 1). This screening process led to the exclusion of 3876 articles and identification of 98 articles for full-text review. Three researchers (SGSS, DN, and VK) independently read the full texts of these 98 articles.

When recommendations differed between reviewers at the title, abstract, and full-text review stages, another reviewer (HCvW) reviewed these articles, and his recommendations to either include or exclude an article were final.

Finally, 92 articles were excluded, and the remaining 6 articles were included in the data extraction (Figure 1). All these 6 studies were included in the narrative synthesis, whereas 5 studies—all clinical trials involving an intervention group and a control group—were included in the meta-analysis (Figure 1). One study with a pre- and postintervention design involving only the intervention group was excluded from the meta-analysis.

### Data Collection Process

For data collection, we used an a priori data extraction template (Tables 1 and 2), which comprised several columns: authors, year, and country of study; study aim or objectives; research design; settings; participants’ characteristics (age, gender, and ethnicity); health or medical condition; sampling method and sample size; participant attrition (numbers and percentages); research methods and data collection tools; interventions (eg, type and tool of digital technology); comparators (eg, alternative intervention, placebo, or care as usual); intervention duration (weeks or months); measurement stages (eg, baseline and follow-up: weeks or months after the baseline); outcomes, result, and findings (eg, loneliness scores, including statistics; eg, mean values, SDs, SEs, and CIs); and study authors’ conclusions [37].

**Table 1:** Characteristics of included studies, participants, sampling methods and sizes, and data collection tools.

Study, country	Quality of evidence^a^ (reviewers’ assessment)	Research design	Settings	Participants				Main health or medical conditions investigated		Sampling method		Sample size				Participant attrition		Research methods or data collection tools
				Age (years)	Gender	Ethnicity					Total		Intervention group	Control group			Loneliness scale used	
Tsai et al (2010) [46], Taiwan	Medium	Quasi-experimental study (NRCT^b^)	Nursing home	Baseline: experimental group: average age 74.2 (SD 10.18); control group: average age 78.48 (SD 6.75)	Male=24 (experimental group=10; control group=14); female=33 (experimental group=14; control group=19)	Not reported (probably all Taiwanese or Chinese)	Loneliness and depression		Purposive		57 baseline; 49 end of study		24 baseline; 21 follow-up	33 baseline; 28 follow-up	8 (5 from control group and 3 from experimental group); attrition rate=14%		UCLA^c^ loneliness scale [47]	
van der Heide et al (2012) [48], The Netherlands	Low	Before and after study (with intervention group only, no control group)	Older home care	Baseline: average age 73.2 (SD 11.8), range 32-90; end of study: average age 73.1 (SD 11.2), range 38-90	Baseline: male=26 (30.2%), female=60 (69.8%), missing values=44; end of study: male=25 (29.4%), female=60 (70.6%), missing values=0	Not reported	Loneliness and safety issues		Convenience		130		130	85=intervention group at the end of study; no control group	45; attrition rate=34.6%		De Jong-Gierveld loneliness scale (score range: 0-11) [49]	
Larsson et al (2016) [50], Sweden	High	Randomized, crossover trial	Living in ordinary housing without any home care services	Range: 61-89, mean 71.2; group 1 (intervention or control group): range 66-89, mean 73.4; group 2 (control or intervention group): range 61-76, mean 69.0	Male=6; female=24, (3 males and 12 females each in group 1 [intervention or control group] and group 2 [control or intervention group])	Not reported (probably all Swedes)	Loneliness		Randomized (after recruitment)		30		15 baseline, 14 follow-up	15 baseline, 14 follow-up	2 (1 participant each from intervention and control groups); attrition rate=6.7%		UCLA loneliness scale [51], the Swedish version (20 items, score range 20-80) [52]	
Czaja et al (2018) [45], United States	High	Multisite randomized controlled trial	Living in independent housing in the community	Baseline: total sample mean 76.15 (SD 7.4), range: 65-98; intervention (PRISM^d^ System) group: mean 76.9 (SD 7.3); control (Binder^e^) group: mean 75.3 (SD 7.4)	Baseline: female=78% (number not reported), male=22% (number not reported); PRISM or intervention group: female 79.3% (n=119); Binder (control) group: female 76.7% (n=115)	Baseline: White=54% and non-White=46%; PRISM or intervention group: non-White or Hispanic=8% (n=12); Binder group: non-White or Hispanic=10% (n=15)	Social isolation, social support, loneliness, and well-being		Randomized		300 (150 in each intervention [PRISM] group and control [Binder] group)		150 baseline; 134 follow-up	150 baseline; 118 follow-up	56 (45 at 6 months and 11 at 12-month follow-up); attrition rate=18.7%		UCLA loneliness scale (score range 20-80) [51]	
Morton et al (2018) [53], United Kingdom	High	2 (condition: training, control)×2 (population: domiciliary, residential)×2 (time: baseline, follow-up) design	Receiving care in own home or supported housing in the community (*domiciliary care*) or residential care in care homes	Female: mean 80.71 (SD 8.77); male: data not reported	Follow-up: total=76; female=50, male=26	Not reported	Well-being and social support		Randomized		97 baseline; 76 follow-up		53 baseline; 44 follow-up	44 baseline; 32 follow-up	21 (9 experimental group; 12 control group); attrition rate=21.6%		UCLA loneliness scale (score range 20-80) [51]	
Jarvis et al (2019) [54], South Africa	High	Randomized control study	Inner-city residential; NGO^f^ care facilities for resource-restricted older people (aged ≥60 years)	Mean 74.93 (SD 6.41); range 61-87	Baseline: male=6 (18.8%), female=26 (81.2%)	Mostly Asian (of Indian origin), numbers not reported	Maladaptive cognitions and loneliness		Randomized		Baseline=32 (intervention group=15, control group=17), final=29 (intervention group=13, control group=16)		15 baseline; 13 follow-up	17 baseline; 16 follow-up	3 (2 intervention group, 1 control group); attrition rate=15.6%		De Jong-Gierveld loneliness scale (score range 0-11) [49]	

^a^Quality of evidence grades: high (we are very confident that the true effect lies close to that of the estimate of the effect), moderate (we are moderately confident in the effect estimate: the true effect is likely to be close to the estimate of the effect, but there is a possibility that it is substantially different), low (our confidence in the effect estimate is limited: the true effect may be substantially different from the estimate of the effect), and very low (we have very little confidence in the effect estimate; the true effect is likely to be substantially different from the estimate of effect).

^b^NRCT: nonrandomized clinical trial.

^c^UCLA: University of California, Los Angeles.

^d^PRISM: Personal Reminder Information and Social Management.

^e^Binder refers to a group of participants who received a notebook with printed content similar to the Personal Reminder Information and Social Management System.

^f^NGO: nongovernmental organization.

**Table 2:** Interventions, outcomes, measurements, results, and conclusions of included studies.

Study	Interventions	Comparators	Intervention duration	Follow-up duration	Outcomes: loneliness scores by measurement stages, mean (SD)						Results or findings		Conclusion by the authors of the study
					Baseline	3 months	4 months	6 months	12 months				
Tsai et al (2010) [46]	Videoconferencing (using either MSN^a^ messenger or Skype)	Regular care	3 months	3 months	Intervention group=50.58 (SD 11.16); control group=46.55 (SD 9.07)	Intervention group=47.33 (SD 13.50); control group=46.68 (SD 9.08)	Not measured	Not measured	Not measured	Loneliness: intervention group mean: baseline 50.58 (SD 11.16), 1 week 49.75 (SD 11.79), and 3 months 47.33 (SD 13.50); control group mean: baseline 46.55 (SD 9.07), 1 week 47.06 (SD 8.75), and 3 months 46.68 (SD 9.08); differences between groups were compared at 3 points (baseline, 1 week, and 3 months) using multiple linear regression of the generalized estimating equations. Unadjusted or fixed effect size of effectiveness of videoconferencing intervention (videoconference vs control): at 1 week was β=−1.21, SE 0.50, *χ*^2^=5.9, *P*=.02 and at 3 months β=−2.84, SE 1.28, *χ*^2^=4.9, *P*=.03		Videoconferencing alleviates depressive symptoms and loneliness in older residents in nursing homes	
van der Heide et al (2012) [48]	CareTV including Caret duplex video or voice network	No control group and no comparator	12 months	12 months	Intervention group=5.97 (SD 2.77); no control group	Not measured	Not measured	Not measured	Intervention group=4.02 (SD 3.91); no control group	Group-level total loneliness: inclusion stage: mean 5.97 (SD 2.77), end of study: mean 4.02 (SD 3.91), *P*=.001; individual-level total loneliness: total loneliness decreased in 54 out of 85 participants (equally lonely 11, more lonely 20, and less lonely 54 individual participants)		CareTV intervention decreased the feeling of loneliness in the participants; however, participants were feeling moderate loneliness at the end of the study	
Larsson et al (2016) [50]	SIBAs^b^, that is, social activities via social websites	No comparator intervention reported	3 months	34 weeks (exposure for 3 months to each group)	Group 1 (I/C^c^ group)=45.53 (SD 7.41); group 2 (C/I^d^ group)=43.93 (SD 8.61)	Group 1 (I/C group)=42.43 (SD 7.44); group 2 (C/I group)=41.93 (SD 8.82)	Not measured	3 months after cross over: group 1 (I/C group, no intervention)=42.0 (SD 7.34); group 2 (C/I group, intervention introduced)=39.50 (SD 10.42)	Not measured	Percentage change between time 2 and time 1: group 1: mean score 0.07% (SD 0.07), *P*=.003; group 2: mean score: 0.05% (SD 0.09), *P*=.049; percentage change between time 3 and time 1: group 1: mean score 0.08% (SD 0.08); group 2: mean score 0.09% (SD 0.13); comparison of pre and postintervention scores: group 1, *P*=.003 and group 2, *P*=.049		SIBA interventions have the potential to reduce experiences of loneliness in socially vulnerable older adults.	
Czaja et al (2018) [45]	PRISM^e^ system	A notebook with printed content similar to that within the PRISM (intervention) group: included a Lenovo *Mini Desktop* PC with a keyboard, mouse (or trackball for those who were unable to control a mouse), a 19″ LCD^f^ monitor, the PRISM software app, a printer, and internet	12 months	12 months	Intervention (PRISM) group=39.8 (SD 9.7); control (Binder^g^) group=40.2 (SD 10.3)	Not measured	Not measured	Intervention (PRISM) group=37.8 (SD 9.54); control (Binder) group=40 (SD 10.62)	Intervention (PRISM) group=36.9 (SD 9.16); control (Binder) group=38.43 (SD 9.37)	Baseline: loneliness PRISM group: mean score 39.8 (SD 9.7); Binder group: mean score 40.2 (SD 10.3), follow-up at 6 months: PRISM group 37.8, Binder group 39.6; follow-up at 12 months: PRISM group 36.9, Binder group 38.3		Technology-based apps such as the PRISM system may enhance social connectivity and reduce loneliness among older adults.	
Morton et al (2018) [53]	EasyPC—a customized computer platform with a simplified touch-screen interface	Care as usual plus regular carer visits	3 months	4 months	Intervention (training) group (total of residential and domiciliary groups)=1.92 (SE 0.10, SD 0.73); control group (total of residential and domiciliary groups)=2.08 (SE 0.12, SD 0.80)	Not measured	Intervention (training) group (total of residential and domiciliary groups)=1.86 (SE 0.10, SD 0.66); control group (total of residential and domiciliary groups)=2.12 (SE 0.11, SD 0.62)	Not measured	Not measured	Loneliness scores mean: intervention (training) group: residential group: time 1=1.95 (SE 0.16), time 2=1.92 (SE 0.16), domiciliary group: time 1=1.89 (SE 0.13), time 2=1.79 (SE 0.13), total time 1=1.92 (SE 0.10), time 2=1.86 (SE 0.10); control group: residential group: time 1=2.13 (SE 0.18), time 2=2.20 (SE 0.17), domiciliary group: time 1=2.02 (SE 0.16), time 2=2.05 (SE 0.15), total time 1=2.08 (SE 0.12) and time 2=2.12 (SE 0.11)		Internet access and training can support the self and social connectedness of vulnerable older adults and contribute positively to well-being.	
Jarvis et al (2019) [54]	Living In Network-Connected Communities WhatsApp group for low-intensity cognitive behavioral therapy	Usual care, a separate WhatsApp group (Living In Network-Connected Communities 2)	3 months	4 months	Not measured	Intervention group=2.31 (SD 1.49); control group=2.47 (SD 2.1)	Intervention group=1.38 (SD 1.33); control group=4.0 (SD 1.32)	Not measured	Not measured	Loneliness levels: total=baseline−intervention on time 1−intervention on time 2.; *χ*^2^=14.6; *P*=.001		Low-intensity cognitive behavioral therapy mobile health supported by the social networking platform of WhatsApp (Living In Network-Connected Communities) showed significant improvements in loneliness and maladaptive cognitions.	

^a^MSN: Microsoft Network.

^b^SIBA: social internet-based activity.

^c^I/C: intervention/control.

^d^C/I: control/intervention.

^e^PRISM: Personal Reminder Information and Social Management.

^f^LCD: liquid-crystal display.

^g^Binder refers to a group of participants who received a notebook with printed content similar to the Personal Reminder Information and Social Management System.

SGSS and DN independently extracted data from all included studies (n=6) using the data extraction template (Tables 1 and 2) and resolved discrepancies in the extracted data with discussion and agreement. Data extraction forms were compared and contrasted, thereby avoiding bias and reducing errors in the data extraction process [55]. We extracted aggregated data at the study level as much as possible with respect to the intervention, which is imperative for the reproducibility of effective interventions [56,57]. Following suggestions for reporting data once from studies with duplicate and multiple publications [55], we extracted and reported data only once [50] from a research study with multiple publications [50,58].

### Data Synthesis and Reporting

We report both a narrative synthesis (narrative summary) and a statistical (quantitative) synthesis (meta-analysis) of our review, as suggested for reporting of a systematic review on effectiveness [59]. In the narrative synthesis, we have included all 6 studies and reported their characteristics, including the study design, settings, sample sizes, data collection methods, participants, interventions, comparators, outcome measurements, and study conclusions.

In the meta-analysis, we have included 5 studies and pooled extracted data on loneliness measured by continuous loneliness scales, that is, the University of California, Los Angeles (UCLA) loneliness scale [47,51] in 4 studies and the De Jong-Gierveld loneliness scale [49] in 2 studies (Table 1). Loneliness scores at baseline and follow-up were reported as the mean values and SDs in 5 studies, whereas 1 study reported mean scores with SEs. For the latter study, we calculated SDs from SEs using a formula suggested in the Cochrane guidelines [60].

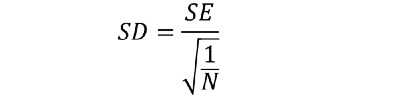



In meta-analysis, the standardized mean difference (SMD) as a summary statistic for reporting continuous data has been suggested for studies that assess the same outcome but use different scales to measure the outcome [60]. In RevMan (The Cochrane Collaboration), the SMD is the effect size known as Hedges (adjusted) g, which is akin to Cohen *d* and includes an adjustment for small sample size bias [60]. More importantly, the generalizability of the SMD statistic is more than the mean difference statistic in a meta-analysis [61].

In our review, the main outcome, that is, loneliness, was measured using different loneliness scales, which included the UCLA loneliness scale (score range 20-80) [47,51] and the De Jong-Gierveld loneliness scale (score range 0-11) [49]. Although these 2 loneliness scales have commonalities such as self-reporting measures and focus on the functional dimension of social relationship and the degree of subjectivity covering perceived availability, adequacy, and emotions or feelings, they differ from each other in other aspects, such as the content and formulation of items or questions included in the scales [62]. In addition, the 2 measures have different number of items or questions, rating options, scoring methods, total scores, and scale versions (for details, refer to the studies by Russell [51] and Russell et al [47] for the UCLA loneliness scale and the studies by De Jong-Gierveld and Tilburg [49] and De Jong-Gierveld and Kamphuls [63] for the De Jong-Gierveld loneliness scale).

The Cochrane guidelines for systematic reviews and meta-analysis [60] suggest that different study designs should not be combined in a meta-analysis because it can increase heterogeneity, and studies with repeated measurements at different follow-up periods cannot be combined without a unit of analysis error.

We extracted data from 6 studies, which included 5 clinical trials [45,46,50,53,54] and 1 pre-post study [48]. Therefore, we included similar study designs, that is, clinical trials in the meta-analysis, and conducted separate meta-analyses based on the same follow-up measurement periods in the clinical trials. Therefore, we performed a separate meta-analysis for each follow-up, that is, measurements at 3, 4, and 6 months after the intervention. In addition, we ran meta-analyses when there were at least two or more studies for the same outcome or the same follow-up period [64]. Therefore, we did not conduct a meta-analysis for follow-up measurements at 12 months reported in 2 studies because they involved different study designs, that is, a randomized controlled trial (RCT) with intervention and control groups [45] and a pre- and postintervention study with only intervention group [48]. This was done to avoid an increase in the heterogeneity [60] and overestimation of the effect of intervention in the absence of a control group [65] in the pre- and postintervention study [48]. We did not perform a meta-analysis for the pre-post study [48] because meta-analysis cannot be performed with only 1 study [60].

We calculated the SMDs from the extracted data, that is, loneliness mean scores with SD and sample sizes in the intervention and control groups at follow-up measurements at 3 months and beyond. For conducting meta-analysis, we used the Cochrane Review Manager (RevMan) software, version 5.3.5 [66]. In the meta-analysis, we used the random effects model as the statistical model because we hypothesized that the true effect sizes between studies would vary [67,68] due to differences in the methodological and clinical characteristics between studies [69], such as differences in the sample sizes, participant numbers and characteristics, intervention types and durations, and follow-up measurement times. We did not conduct sensitivity analyses because of the small number of studies in the meta-analyses at each follow-up point [64].

### Assessment of Research Quality, Bias, and Heterogeneity

We assessed the quality of research by applying the GRADE (Grading of Recommendations Assessment, Development and Evaluation) approach [70].

We assessed the risk of bias by focusing on 5 domains: the evaluation of sequence generation, allocation concealment, blinding (outcome assessors), incomplete data, selective outcome reporting, and assessing other biases using the Cochrane guidelines [60]. In a meta-analysis, publication bias can be assessed with a graphical method using funnel plots [60,71] and statistical methods such as the Egger test [60]; however, both methods require at least 10 studies in the meta-analysis [60]. When the number of studies is small, the Egger test has low power and fails to differentiate chance from real asymmetry [60]. Similarly, assessing publication bias using funnel plots with fewer studies would be of very limited usefulness because it would be difficult to spot the publication bias. As we had a maximum of 3 studies in a meta-analysis, we could not check the publication bias with either method.

We checked heterogeneity, that is, variation in study outcomes or intervention effect sizes between studies, by the Cochran Q test with a significance level of *ρ<*0.10 [72,73] because of the low power of the test in a meta-analysis with very few studies or studies with small sample sizes [74]. We calculated I^2^ statistics to determine the magnitude of heterogeneity (ie, the proportion of variance in the true effect sizes) between studies [28]. We considered I^2^ values of 25%, 50%, and 75% as low, moderate, and high heterogeneity between studies, respectively [75].

### Summary Measures

We report the findings of meta-analyses using SMDs with 95% CIs as a statistical summary, with the forest plots [60].

## Results

### Narrative Synthesis

Findings about the characteristics of the studies, including the study designs, settings, participants, interventions, comparators, sample sizes, participant attrition, and data collection methods or tools used, are presented in Table 1. The interventions, comparators, follow-up durations, outcomes or measurement scores, results, and conclusions of the included studies are given in Table 2.

#### Study Selection

Searches of PubMed, MEDLINE, CINAHL, Embase, and Web of Science generated a total of 4939 articles (Figure 1), of which 6 studies met the predefined eligibility criteria. All 6 studies were included in the narrative synthesis, 5 clinical trials with the intervention and control groups were included in the meta-analysis, and only 1 study with a pre-post design involving only the intervention group was excluded from the meta-analysis.

#### Study Participants

The total number of participants enrolled in all 6 included studies was 646 (mean 108, SD 102; median 77, IQR 32-130). Studies varied in total sample sizes (mean 108, SD 102; range 30-300), and the sample sizes of the intervention and control groups also varied at both the baseline and follow-up measurements across the studies (Table 1). The attrition rate also varied between studies (range 7%-35%; mean 19%, SD 10%).

Participants’ average age was between 73 and 78 years (SD 6-11). Total enrolled participants included 66.1% (427/646) women and 23.8% (154/646) men, whereas for 10.1% (65/646) of participants, no information about their gender was available. Studies varied in the proportion of male and female participants (female: mean 66%, SD 16%; range 46%-81%; male: mean 25%, SD 9%; range 19%-42%). Only 2 studies reported on participants’ ethnicity—White (54%) and non-Whites (46%) in the US study [45] and mostly Asian Indians (no numbers reported) in the South African study [54].

#### Study Characteristics

A total of 4 studies were RCTs [45,50,53,54], 1 study was a nonrandomized clinical trial [46], and 1 was a pre- and posttest (before and after) study with intervention group only (no control group) [48] (Table 1).

#### Study Settings

A total of 4 studies were conducted in developed countries, namely, the Netherlands [48], the United Kingdom [53], the United States [45]**,** and Sweden [50]. Two studies were undertaken in developing countries, namely, Taiwan [46] and South Africa [54].

The settings included living in independent housing in the community [45]; living in ordinary housing without any home care services [50]; receiving care in their own home or supported housing in the community (*domiciliary care*), or receiving care in residential care homes [53], residential care facilities for older people [54], nursing homes [46], and older home care [48].

Participants were selected by random sampling in 66.7% (4/6) of studies [45,50,53,54], whereas the other 33.3% (2/6) studies used purposive [46] and convenience [48] sampling each.

#### Digital Technology Interventions

DTIs included social internet-based activities, that is, social activities via social websites [50], videoconferencing [46], customized computer platforms with simplified touch-screen interfaces [53], personal reminder information and social management systems [45], WhatsApp groups [54], and video or voice networks [48].

#### Duration of the Intervention and Measurement of the Main Outcome Measure

The duration of the intervention was 3 months in 4 studies [46,50,53,54] and 12 months in 2 studies [45,48]. The main outcome measure, that is, loneliness, was measured at the baseline and multiple follow-up times, which included 3 months in 3 studies [46,50,54], 4 months in 2 studies [53,54], 6 months in 2 studies [45,50], and 12 months in 2 studies [45,48].

The loneliness measurement tools used were the UCLA loneliness scale [47,51], which was applied in 4 studies [45,46,50,53], and the De Jong-Gierveld loneliness scale [49,76], which was used in 2 studies [48,54]. Table 2 presents loneliness scores measured in the intervention and control groups, if any, at baseline and follow-ups.

Narrative synthesis showed that there was a reduction in loneliness in the intervention groups at the follow-ups compared with baseline (Table 2). A statistical summary of the loneliness measurements in the intervention and control groups at the follow-ups is reported in the *Meta-analysis* section.

### Meta-analysis

We conducted 3 meta-analyses, 1 each for follow-up measurements at 3, 4, and 6 months, involving 3, 2, and 2 studies, respectively.

#### Meta-analysis for Follow-up at 3 Months

**Figure 2:**
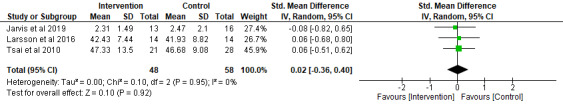
Forest plot of standardized mean differences for loneliness at the 3-month follow-up (digital technology intervention vs control).

Three studies [46,50,54] involving 106 participants with follow-up measurements at 3 months were entered into a meta-analysis, which showed a very small reduction in loneliness in favor of the control (SMD 0.02; 95% CI −0.36 to 0.40), but it was not statistically significant (Z=0.10; *P*=.92). The heterogeneity between studies was not statistically significant (τ^2^=0.00; χ^2^_2_=0.1; *P*=.95; I^2^=0%; Figure 2).

#### Meta-analysis for Follow-up at 4 Months

**Figure 3:**
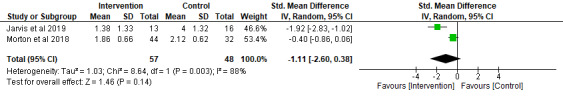
Forest plots of standardized mean differences for loneliness at the 4-month follow-up (digital technology intervention vs control).

Two studies [53,54] involving 105 participants with 4 month follow-up were entered into a meta-analysis, which revealed a large reduction in loneliness in favor of the intervention (SMD −1.11; 95% CI −2.60 to 0.38), but it was not statistically significant (Z=1.46; *P*=.14). There was a statistically significant high heterogeneity between studies (τ^2^=1.03; χ^2^_1_=8.8; *P*=.003; I^2^=88%; Figure 3).

#### Meta-analysis for Follow-up at 6 Months

**Figure 4:**
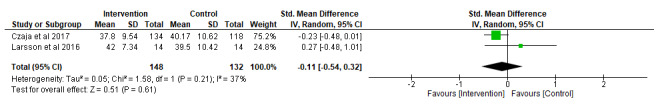
Forest plots of standardized mean differences for loneliness at the 6-month follow-up (digital technology intervention vs control).

A meta-analysis involving 2 studies [45,50] with 280 participants with 6 month follow-up showed a very small reduction in loneliness in favor of the intervention (SMD −0.11; 95% CI −0.54 to 0.32), but it was not statistically significant (Z=0.51; *P*=.61). There was moderate heterogeneity between studies, but it was not statistically significant (τ^2^=0.05; χ^2^_1_=1.6; *P*=.21; I^2^=37%; Figure 4).

### Risk of Bias

**Figure 5:**
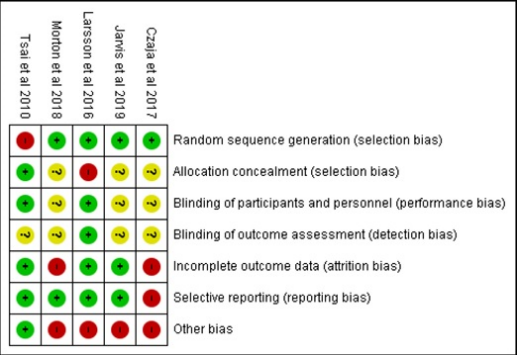
Risk of bias summary. Review authors’ judgments about risk of bias in included studies: Czaja et al, 2017 [45], Tsai et al 2010 [46], Larsson et al, 2016 [50], Morton et al, 2018 [53], and Jarvis et al, 2019 [54].

**Figure 6:**
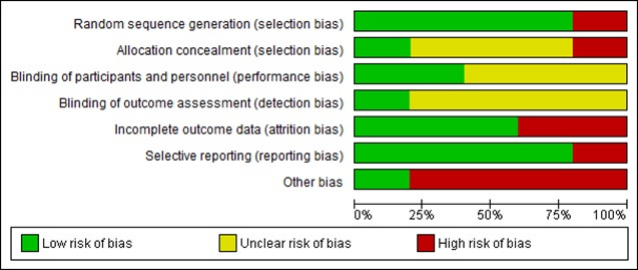
Risk of bias graph. Review authors’ judgments about each risk of bias item are presented as percentages across all included studies.

The risk of bias assessment, that is, the risk of bias graph and risk of bias summary are presented in Figure 5 and Figure 6, respectively. A high risk of bias was noted in the attrition bias and other biases; an unclear risk of bias was detected in the blinding of outcome assessment, allocation concealment, and blinding of participants and personnel; and a low risk of bias was observed, especially, in the random sequence generation and selective reporting (Figure 6). In addition, most studies reported only within-group changes and not between-group comparisons of change, which may suggest a weak quality of the reporting of results and the analysis in these studies (Table 2).

### Quality of Evidence

**Figure 7:**
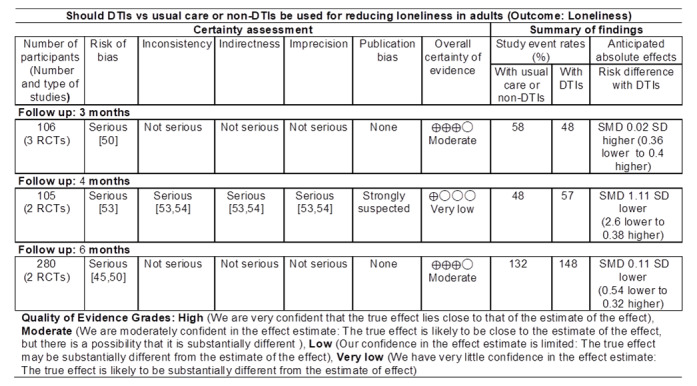
GRADE (Grading of Recommendations Assessment, Development and Evaluation) quality of evidence summary. DTI: digital technology intervention; RCT: randomized controlled trial; SMD: standardized mean difference.

The quality of evidence was moderate, very low, and moderate in meta-analyses involving 3 [46,50,54], 2 [53,54], and 2 studies [45,50], with follow-up at 3, 4, and 6 months, respectively (Figure 7).

## Discussion

### Principal Findings

To determine whether DTIs are effective in reducing loneliness in adults, we appraised peer-reviewed empirical research involving the application of DTIs in adults with loneliness. Our systematic review provides a narrative summary (qualitative synthesis) as well as a meta-analysis (statistical synthesis) of the findings. The narrative summary of 6 studies included in our review showed a reduction in loneliness in the intervention groups at follow-up compared with baseline (Table 2). However, our meta-analysis of 5 clinical trials with follow-up measurements at 3, 4, and 6 months showed no statistically significant pooled effect estimates as SMDs, the preferred method for summarizing effects on continuous outcomes such as loneliness. Although not statistically significant, the summary effect size at the 4-month follow-up (Figure 3) was better than the effect size at the 3-month follow-up (Figure 2) and the 6-month follow-up (Figure 4).

Our meta-analysis also revealed that CIs of the summary effects of 2 studies, that is, the studies by Larsson et al [50] and Tsai et al [46], were very wide, and the SMDs from these studies were more in favor of the control group than the intervention group (Figure 2). Thus, the wide range of CIs of the summary effects in these studies leave room for uncertainty about the beneficial effect of DTIs on measures of loneliness.

Overall, the findings of our meta-analysis showed no evidence supporting the effectiveness of DTIs in reducing loneliness in older adults.

### Summary of Evidence

The quality of evidence of the included studies was very low to moderate (Figure 7), and there was a high heterogeneity between studies [53,54] (Figure 2). All the included studies had a high proportion of female participants. Most notably, the total number of participants was low, especially in 2 studies [50,54], and the sample sizes were reduced further due to a high attrition rate in some studies [45,53]. The types and methods of DTIs varied between studies (Table 2), which were conducted in diverse settings (Table 1). Studies were conducted in 6 different countries: the United States, the United Kingdom, Sweden, the Netherlands (these four countries have individualistic cultures), Taiwan, and South Africa (these two countries have collectivist cultures; Table 1). In addition, despite our inclusion criteria of age 18 years and above, the selected studies more commonly involved older people with an average age of 70 years and above.

Loneliness is influenced by culture [77-79], gender [78], and age [5,78], and these factors could have contributed to the pooled estimates being not statistically significant in our meta-analysis. In addition, differences in participants, especially in terms of age, gender, and culture as well as varied types of DTIs, could have contributed to the heterogeneity observed, especially in the meta-analysis with the 4 month follow-up involving 2 studies [53,54], which differed from each other on different parameters, especially the study designs, settings, participants, interventions, and loneliness measurement scales used (Tables 1 and 2).

There are limited published meta-analyses on technological interventions for tackling loneliness, and a few existing studies have covered literature published up to 2009 [28] and 2011 [30]. Our review and meta-analysis included the latest evidence published between January 1, 2010, and July 31, 2019. We did not replicate the findings of earlier meta-analyses that reported evidence suggesting that technological interventions resulted in decreased loneliness [28,30]. For example, a meta-analysis by Choi et al [30] reported statistically significant evidence suggesting that the internet and computers reduce loneliness. However, they [30] focused on older adults with depression and included the internet and computers only as technological interventions, whereas we included different types of DTIs, and our population of interest was adults of all age groups (≥18 years). In addition, the meta-analysis by Choi et al [30] included studies (n=5) with different follow-up periods (3-6 months), but they did not report which follow-up measurements were included in their meta-analysis. In our meta-analysis, we conducted separate meta-analyses for measurements at different follow-up periods, that is, 3, 4, and 6 months, as suggested by the Cochrane guidelines [60].

A meta-analysis by Masi et al [28] also reported that technological interventions reduce loneliness, which was more in pre-post studies and nonrandomized studies than in RCTs. However, they included studies with technology and nontechnology-based interventions [28], whereas we focused on studies with DTIs only. In addition, Masi et al [28] did not report how they analyzed measurements at different follow-up periods, whereas we did not combine measurements at different follow-up times, as suggested by the Cochrane guidelines [60]. Nonetheless, Masi et al [28] concluded that technology is yet to be capitalized for loneliness.

Interestingly, our findings provide new insights about DTIs and loneliness. Our meta-analysis showed no statistically significant reduction in loneliness in the intervention groups compared with the control groups at the 3 -, 4-, and 6-month follow-ups. Thus, our findings show no evidence supporting the effectiveness of DTIs in reducing loneliness in older adults, which goes beyond the findings of a recent Cochrane review that reported no evidence of video calls being effective in reducing loneliness in older adults [73].

In addition, our findings refute and contradict a commonly held view that digital technology can solve the problem of loneliness, especially in older people. Nonetheless, digital technologies provide tools and means that facilitate social connection [80], which may help in reducing loneliness for a limited period because the effects of DTIs are short-lived [81]. This may be because digital technologies do not provide real human interaction [80] and cannot replace human contact [45]; thus, they do not reduce social disconnectedness in real life [82] on a long-term basis.

Nonetheless, a review has reported that some nontechnological interventions are effective in reducing loneliness in older people [83], but these interventions require a meta-analytic evaluation. In addition, a recent meta-analysis [84] reported moderate evidence of the effectiveness of a range of social, emotional, and psychological interventions, delivered through technological and nontechnological means, in reducing loneliness in young people aged 3-25 years; however, the analyzed studies had limitations. Therefore, further research is required.

### Limitations

Our study had some limitations: the inclusion of only 6 studies with heterogeneous sets of results and the minimum intervention duration of 3 months, which could have resulted in the inclusion of a small number of studies and possible exclusion of potential studies that would have provided useful evidence.

In addition, we could not conduct subgroup and meta-regression analyses due to the very limited number of studies (n=5) in the meta-analysis and lack of data on loneliness by participants’ demographic characteristics. In addition, our study might be narrow because we excluded some studies [44,85-91], which met the technology criterion such as the use of robots, sensors, digital speakers, and apps but did not meet other selection criteria. Thus, our study may be limited to studies about social interactions and connectedness using digital technology tools.

Moreover, another limitation of our review could be the use of a meta-analysis based only on follow-up data. For example, a study by Tsai et al [46] in the 3-month follow-up meta-analysis had an SMD of 0.06 with a 95% CI of −0.8 to 0.65 (*P*=.03; Figure 2), which may suggest that these studies may have had higher power to show a difference compared with baseline loneliness.

As recommendations for future research, we suggest that researchers involved in trials agree on a common measure of loneliness and consider reporting of results in a standardized way, which will allow pooling of baseline-adjusted estimates of the treatment effect rather than differences in follow-up means.

### Conclusions

Our meta-analysis showed no evidence supporting the effectiveness of DTIs in reducing loneliness in older adults. Therefore, there is a need for further research involving RCTs [50] with larger sample sizes and longer duration of interventions and follow-up measurement periods. Future research may apply inclusive research designs using a combination of digital apps, including robots, sensors, and social connecting apps, by involving adults aged 45-65 years, as this segment of the population is more likely to be more technology savvy and digital interventions might be more effective in this age group. Future research might also target ethnic minority communities and specific groups such as lesbian, gay, bisexual, and transgender people where loneliness is common [8,92].

## Appendix

Multimedia Appendix 1 Literature searches.

## Abbreviations

DTI: digital technology intervention
GRADE: Grading of Recommendations Assessment, Development and Evaluation
PICO: participant, intervention, comparator, and outcome
PRISMA: Preferred Reporting Items for Systematic Reviews and Meta-Analyses
RCT: randomized controlled trial
SMD: standardized mean difference
UCLA: University of California, Los Angeles
