# Supplementary material for: Evaluation of the Effectiveness of Digital Technology Interventions to Reduce Loneliness in Older Adults: Systematic Review and Meta-analysis
Source: J Med Internet Res. 2021 Jun 4;23(6):e24712. doi: 10.2196/24712 (PMC8214187; doi:10.2196/24712)
Supplement: Multimedia Appendix 1 [file jmir_v23i6e24712_app1.docx]

**Multimedia Appendix 1. Literature searches**

| **Search Query** | **DATABASES SEARCHED** | | | | |
| --- | --- | --- | --- | --- | --- |
|  | **PubMed** | **EMBASE** | **Medline** | **CINAHL** | **Web of Science** |
| ***Subject Headings*** |  | ***(via OVID)*** | ***(via OVID)*** | ***(via EBSCOHOST)*** |  |
| Loneliness | Yes | Yes | Yes | Yes | No subject headings |
| Technology | No | No | No | Yes | No subject headings |
| Robotics | Yes | Yes | Yes | Yes | No subject headings |
| Internet | Yes | Yes | Yes | Yes | No subject headings |
| Social Media | Yes | Yes | Yes | Yes | No subject headings |
| Telephone | Yes | Yes | Yes | Yes | No subject headings |
| Smartphone | Yes | Yes | Yes | Yes | No subject headings |
| Computer | Yes | Yes | Yes | Yes | No subject headings |
| Computer handheld | Yes | No | Yes | Yes | No subject headings |
| Personal digital assistant | No | Yes | No | No | No subject headings |
| Web (world Wide Web) | No | No | No | Yes | No subject headings |
| ***Phrases / text*** |  |  |  |  |  |
| Loneliness **OR** lonely **OR** social isolation [Title/Abstract] | Yes | Yes | Yes | Yes | **Searched as topics** (includes: Title, Abstract, Author Keywords, Keywords Plus) |
| **AND** |  |  |  |  |  |
| Digital* **OR** technolog* **OR** sensor* **OR** robot* **OR** internet **OR** social media **OR** smartphone* **OR** smart phone* OR telephone* **OR** phone **OR** online **OR** ipad* **OR** computer* **OR** electronic* **OR** Web [Title/Abstract] | Yes | Yes | Yes | Yes | digital* OR technolog* OR sensor* OR robot* OR internet OR social media OR smartphone* OR online OR ipad* OR computer* OR electronic* OR Web [**Searched as topics**] |
| **Filters** |  |  |  |  |  |
| **Publication years:** 2010-2019 | Yes | Yes | Yes | Yes | Yes |
| **Language:** English | Yes | Yes | Yes | Yes | Yes |
| **Species:** Humans | Yes | Yes | Yes | Yes | N/A |
| **Document types:** | Clinical Trial, Controlled Clinical Trial, Evaluation Studies, Journal Article, Meta-Analysis, Observational Study, Randomized Controlled Trial, Review, Systematic Reviews | Trials (all) | Clinical Trial, Evaluation Studies Journal Article Meta-Analysis Observational Study Review Systematic Reviews | Clinical Trial, Journal Article, Meta-Analysis, Randomized Controlled Trial, Research, Review Systematic Review | Articles, Reviews |

**PUBMED SEARCHES**

(("Loneliness"[Mesh]) OR (((loneliness[Title/Abstract]) OR lonely[Title/Abstract]) OR social isolation[Title/Abstract])) AND (("Robotics"[Mesh]) OR ("Internet"[Mesh]) OR ("Social Media"[Mesh]) OR ("Smartphone"[Mesh]) OR ("Telephone"[Mesh]) OR ("Computers, Handheld"[Mesh]) OR ("Computers"[Mesh]) OR (((((((((((((digital*[Title/Abstract]) OR technolog*[Title/Abstract]) OR sensor*[Title/Abstract]) OR robot*[Title/Abstract]) OR internet[Title/Abstract]) OR social media[Title/Abstract]) OR phone*[Title/Abstract]) OR telephone*[Title/Abstract]) OR online[Title/Abstract]) OR ipad*[Title/Abstract]) OR computer*[Title/Abstract]) OR electronic*[Title/Abstract]) OR web[Title/Abstract]))

("Loneliness"[Mesh] OR ((loneliness[Title/Abstract] OR lonely[Title/Abstract]) OR social isolation[Title/Abstract])) AND ("Robotics"[Mesh] OR "Internet"[Mesh] OR "Social Media"[Mesh] OR "Smartphone"[Mesh] OR "Telephone"[Mesh] OR "Computers, Handheld"[Mesh] OR "Computers"[Mesh] OR (((((((((((((digital[Title/Abstract] OR digital'[Title/Abstract] OR digital''[Title/Abstract] OR digital's[Title/Abstract] OR digital1[Title/Abstract] OR digitala[Title/Abstract] OR digitalassets[Title/Abstract] OR digitalb[Title/Abstract] OR digitalcell[Title/Abstract] OR digitalcellsorter[Title/Abstract] OR digitalcommunication[Title/Abstract] OR digitaldiagnost[Title/Abstract] OR digitaldlsorter[Title/Abstract] OR digitaldna[Title/Abstract] OR digitale[Title/Abstract] OR digitaleae[Title/Abstract] OR digitalel[Title/Abstract] OR digitalemia[Title/Abstract] OR digitalemia's[Title/Abstract] OR digitalemic[Title/Abstract] OR digitalen[Title/Abstract] OR digitaler[Title/Abstract] OR digitalera[Title/Abstract] OR digitales[Title/Abstract] OR digitalface[Title/Abstract] OR digitalfiltering[Title/Abstract] OR digitalfishlibrary[Title/Abstract] OR digitalgia[Title/Abstract] OR digitalglobe[Title/Abstract] OR digitalhealth[Title/Abstract] OR digitalhealtheurope[Title/Abstract] OR digitalhealthscore[Title/Abstract] OR digitalhub[Title/Abstract] OR digitalhub's[Title/Abstract] OR digitali[Title/Abstract] OR digitalia[Title/Abstract] OR digitalic[Title/Abstract] OR digitalica[Title/Abstract] OR digitalics[Title/Abstract] OR digitalideae[Title/Abstract] OR digitalidis[Title/Abstract] OR digitalin[Title/Abstract] OR digitalinaemia[Title/Abstract] OR digitaline[Title/Abstract] OR digitalins[Title/Abstract] OR digitalinum[Title/Abstract] OR digitalis[Title/Abstract] OR digitalis'[Title/Abstract] OR digitalis's[Title/Abstract] OR digitalisation[Title/Abstract] OR digitalisconcentration[Title/Abstract] OR digitalisconcentrations[Title/Abstract] OR digitalise[Title/Abstract] OR digitalised[Title/Abstract] OR digitalisglycoside[Title/Abstract] OR digitalisglycosides[Title/Abstract] OR digitalisierten[Title/Abstract] OR digitalisierung[Title/Abstract] OR digitalising[Title/Abstract] OR digitalisintoxication[Title/Abstract] OR digitalisintoxications[Title/Abstract] OR digitalisize[Title/Abstract] OR digitalislike[Title/Abstract] OR digitalism[Title/Abstract] OR digitalispreparations[Title/Abstract] OR digitalisreceptor[Title/Abstract] OR digitality[Title/Abstract] OR digitalizacion[Title/Abstract] OR digitalizada[Title/Abstract] OR digitalizadas[Title/Abstract] OR digitalizaing[Title/Abstract] OR digitalization[Title/Abstract] OR digitalization'[Title/Abstract] OR digitalizations[Title/Abstract] OR digitalize[Title/Abstract] OR digitalized[Title/Abstract] OR digitalizer[Title/Abstract] OR digitalizers[Title/Abstract] OR digitalizes[Title/Abstract] OR digitalizing[Title/Abstract] OR digitalizzata[Title/Abstract] OR digitalizzazione[Title/Abstract] OR digitaljournal[Title/Abstract] OR digitalk[Title/Abstract] OR digitall[Title/Abstract] OR digitallis[Title/Abstract] OR digitallung[Title/Abstract] OR digitally[Title/Abstract] OR digitally'[Title/Abstract] OR digitallyconnected[Title/Abstract] OR digitalmammography[Title/Abstract] OR digitalme[Title/Abstract] OR digitalmed[Title/Abstract] OR digitalmetrade[Title/Abstract] OR digitalmicrograph[Title/Abstract] OR digitalmicrographtrade[Title/Abstract] OR digitalmill[Title/Abstract] OR digitalmlpa[Title/Abstract] OR digitalo[Title/Abstract] OR digitaloid[Title/Abstract] OR digitaloides[Title/Abstract] OR digitaloidites[Title/Abstract] OR digitaloids[Title/Abstract] OR digitalonin[Title/Abstract] OR digitalopyranoside[Title/Abstract] OR digitalopyranosyl[Title/Abstract] OR digitalose[Title/Abstract] OR digitalosid[Title/Abstract] OR digitaloside[Title/Abstract] OR digitalosyl[Title/Abstract] OR digitalotherapy[Title/Abstract] OR digitalpcr[Title/Abstract] OR digitalpt[Title/Abstract] OR digitalradiography[Title/Abstract] OR digitalrectal[Title/Abstract] OR digitalrom[Title/Abstract] OR digitals[Title/Abstract] OR digitalslide[Title/Abstract] OR digitalslidearchive[Title/Abstract] OR digitalspiders[Title/Abstract] OR digitaltf[Title/Abstract] OR digitaltrade[Title/Abstract] OR digitalvhi[Title/Abstract] OR digitalyzed[Title/Abstract]) OR (technolog[Title/Abstract] OR technologeous[Title/Abstract] OR technologia[Title/Abstract] OR technologiae[Title/Abstract] OR technologic[Title/Abstract] OR technological[Title/Abstract] OR technological'[Title/Abstract] OR technologicalchallenge[Title/Abstract] OR technologicalforecasting[Title/Abstract] OR technologicalforesight[Title/Abstract] OR technologicalization[Title/Abstract] OR technologically[Title/Abstract] OR technologically'[Title/Abstract] OR technologicalperspective[Title/Abstract] OR technologics[Title/Abstract] OR technologicus[Title/Abstract] OR technologie[Title/Abstract] OR technologie'[Title/Abstract] OR technologiebewertung[Title/Abstract] OR technologiees[Title/Abstract] OR technologielaan[Title/Abstract] OR technologien[Title/Abstract] OR technologiepark[Title/Abstract] OR technologies[Title/Abstract] OR technologies'[Title/Abstract] OR technologies's[Title/Abstract] OR technologiesare[Title/Abstract] OR technologiesartificial[Title/Abstract] OR technologiesguidance[Title/Abstract] OR technologiesis[Title/Abstract] OR technologiesrome[Title/Abstract] OR technologiessuch[Title/Abstract] OR technologiestm[Title/Abstract] OR technologiestrade[Title/Abstract] OR technologieswe[Title/Abstract] OR technologii[Title/Abstract] OR technologique[Title/Abstract] OR technologiques[Title/Abstract] OR technologis[Title/Abstract] OR technologisation[Title/Abstract] OR technologische[Title/Abstract] OR technologised[Title/Abstract] OR technologising[Title/Abstract] OR technologism[Title/Abstract] OR technologist[Title/Abstract] OR technologist'[Title/Abstract] OR technologist's[Title/Abstract] OR technologists[Title/Abstract] OR technologists'[Title/Abstract] OR technologists'attitudes[Title/Abstract] OR technologists's[Title/Abstract] OR technologization[Title/Abstract] OR technologize[Title/Abstract] OR technologized[Title/Abstract] OR technologizing[Title/Abstract] OR technologlans[Title/Abstract] OR technologly[Title/Abstract] OR technology[Title/Abstract] OR technology'[Title/Abstract] OR technology''[Title/Abstract] OR technology's[Title/Abstract] OR technology1[Title/Abstract] OR technology16[Title/Abstract] OR technology3[Title/Abstract] OR technologyallows[Title/Abstract] OR technologyand[Title/Abstract] OR technologyassisted[Title/Abstract] OR technologyconsider[Title/Abstract] OR technologydagger[Title/Abstract] OR technologyevaluation[Title/Abstract] OR technologyfor[Title/Abstract] OR technologyhave[Title/Abstract] OR technologyhose[Title/Abstract] OR technologyin[Title/Abstract] OR technologyis[Title/Abstract] OR technologyit[Title/Abstract] OR technologymc[Title/Abstract] OR technologypreclinical[Title/Abstract] OR technologys[Title/Abstract] OR technologythat[Title/Abstract] OR technologyto[Title/Abstract] OR technologytrade[Title/Abstract] OR technologytranslation[Title/Abstract] OR technologyuser[Title/Abstract] OR technologyvarious[Title/Abstract])) OR (sensor[Title/Abstract] OR sensor'[Title/Abstract] OR sensor's[Title/Abstract] OR sensor1[Title/Abstract] OR sensor4pri[Title/Abstract] OR sensora[Title/Abstract] OR sensorad[Title/Abstract] OR sensoraid[Title/Abstract] OR sensoral[Title/Abstract] OR sensorale[Title/Abstract] OR sensorally[Title/Abstract] OR sensorand[Title/Abstract] OR sensorant[Title/Abstract] OR sensorapertures[Title/Abstract] OR sensorarray[Title/Abstract] OR sensorart[Title/Abstract] OR sensorband[Title/Abstract] OR sensorbility[Title/Abstract] OR sensorbod[Title/Abstract] OR sensorbox[Title/Abstract] OR sensorcaine[Title/Abstract] OR sensorcatheter[Title/Abstract] OR sensorchip[Title/Abstract] OR sensorchips[Title/Abstract] OR sensorcm[Title/Abstract] OR sensorconsists[Title/Abstract] OR sensordata[Title/Abstract] OR sensordb[Title/Abstract] OR sensordish[Title/Abstract] OR sensore[Title/Abstract] OR sensoready[Title/Abstract] OR sensorealization[Title/Abstract] OR sensored[Title/Abstract] OR sensoredge[Title/Abstract] OR sensoregulator[Title/Abstract] OR sensoremoval[Title/Abstract] OR sensoremoval'[Title/Abstract] OR sensores[Title/Abstract] OR sensorevery[Title/Abstract] OR sensoreverywhere[Title/Abstract] OR sensorex[Title/Abstract] OR sensorexhibited[Title/Abstract] OR sensorfor[Title/Abstract] OR sensorfret[Title/Abstract] OR sensorg[Title/Abstract] OR sensorgraft[Title/Abstract] OR sensorgram[Title/Abstract] OR sensorgrams[Title/Abstract] OR sensorgraphy[Title/Abstract] OR sensorhis[Title/Abstract] OR sensori[Title/Abstract] OR sensoria[Title/Abstract] OR sensoriactuators[Title/Abstract] OR sensoriais[Title/Abstract] OR sensorial[Title/Abstract] OR sensorial'[Title/Abstract] OR sensoriales[Title/Abstract] OR sensorialist[Title/Abstract] OR sensorialite[Title/Abstract] OR sensoriality[Title/Abstract] OR sensorialized[Title/Abstract] OR sensorially[Title/Abstract] OR sensorials[Title/Abstract] OR sensorialy[Title/Abstract] OR sensoribehavioural[Title/Abstract] OR sensoric[Title/Abstract] OR sensorica[Title/Abstract] OR sensorical[Title/Abstract] OR sensorically[Title/Abstract] OR sensoricortical[Title/Abstract] OR sensoricphysiological[Title/Abstract] OR sensorics[Title/Abstract] OR sensoridiscriminative[Title/Abstract] OR sensoriel[Title/Abstract] OR sensorielle[Title/Abstract] OR sensorielles[Title/Abstract] OR sensoriels[Title/Abstract] OR sensories[Title/Abstract] OR sensorif[Title/Abstract] OR sensorigenesis[Title/Abstract] OR sensorii[Title/Abstract] OR sensoril[Title/Abstract] OR sensorilimbic[Title/Abstract] OR sensorily[Title/Abstract] OR sensorimetry[Title/Abstract] OR sensorimortor[Title/Abstract] OR sensorimoter[Title/Abstract] OR sensorimoteur[Title/Abstract] OR sensorimoteurs[Title/Abstract] OR sensorimotility[Title/Abstract] OR sensorimotor[Title/Abstract] OR sensorimotor'[Title/Abstract] OR sensorimotoraffective[Title/Abstract] OR sensorimotoraxonal[Title/Abstract] OR sensorimotorcontrol[Title/Abstract] OR sensorimotorcortical[Title/Abstract] OR sensorimotori[Title/Abstract] OR sensorimotorial[Title/Abstract] OR sensorimotoric[Title/Abstract] OR sensorimotorrhythm[Title/Abstract] OR sensorimotorsystem[Title/Abstract] OR sensorimotory[Title/Abstract] OR sensorimototor[Title/Abstract] OR sensorimotrice[Title/Abstract] OR sensorimotrices[Title/Abstract] OR sensorimotricity[Title/Abstract] OR sensorin[Title/Abstract] OR sensorincludes[Title/Abstract] OR sensorineal[Title/Abstract] OR sensorineral[Title/Abstract] OR sensorineraul[Title/Abstract] OR sensorinerual[Title/Abstract] OR sensorinerural[Title/Abstract] OR sensoriness[Title/Abstract] OR sensorineu[Title/Abstract] OR sensorineual[Title/Abstract] OR sensorineueal[Title/Abstract] OR sensorineumral[Title/Abstract] OR sensorineur[Title/Abstract] OR sensorineura[Title/Abstract] OR sensorineural[Title/Abstract] OR sensorineural'[Title/Abstract] OR sensorineuralhearing[Title/Abstract] OR sensorineuralis[Title/Abstract] OR sensorineurally[Title/Abstract] OR sensorineurals[Title/Abstract] OR sensorineurepithelium[Title/Abstract] OR sensorineurinal[Title/Abstract] OR sensorineurocognitive[Title/Abstract] OR sensorineuroepithelium[Title/Abstract] OR sensorineurological[Title/Abstract] OR sensorineuronal[Title/Abstract] OR sensorineutral[Title/Abstract] OR sensoring[Title/Abstract] OR sensoringeural[Title/Abstract] OR sensorintegrative[Title/Abstract] OR sensorinueral[Title/Abstract] OR sensorinural[Title/Abstract] OR sensorio[Title/Abstract] OR sensorioculomotor[Title/Abstract] OR sensoriomotor[Title/Abstract] OR sensoriomotora[Title/Abstract] OR sensoriomotoras[Title/Abstract] OR sensoriomotriz[Title/Abstract] OR sensorion[Title/Abstract] OR sensorioneural[Title/Abstract] OR sensoriperception[Title/Abstract] OR sensoriperceptual[Title/Abstract] OR sensoriphasic[Title/Abstract] OR sensoriphobia[Title/Abstract] OR sensoriprocessing[Title/Abstract] OR sensoris[Title/Abstract] OR sensorisation[Title/Abstract] OR sensorische[Title/Abstract] OR sensorised[Title/Abstract] OR sensorisensory[Title/Abstract] OR sensoriske[Title/Abstract] OR sensorisomatic[Title/Abstract] OR sensoristasis[Title/Abstract] OR sensoristatic[Title/Abstract] OR sensoristic[Title/Abstract] OR sensoristics[Title/Abstract] OR sensoristrain[Title/Abstract] OR sensoristrain'[Title/Abstract] OR sensorithm[Title/Abstract] OR sensoritmotor[Title/Abstract] OR sensoritopic[Title/Abstract] OR sensoritrigeminal[Title/Abstract] OR sensoritrophic[Title/Abstract] OR sensority[Title/Abstract] OR sensorium[Title/Abstract] OR sensorium'[Title/Abstract] OR sensoriums[Title/Abstract] OR sensorius[Title/Abstract] OR sensorivagal[Title/Abstract] OR sensorization[Title/Abstract] OR sensorize[Title/Abstract] OR sensorized[Title/Abstract] OR sensorized'[Title/Abstract] OR sensorizing[Title/Abstract] OR sensorless[Title/Abstract] OR sensorlike[Title/Abstract] OR sensorlink[Title/Abstract] OR sensorlog[Title/Abstract] OR sensormat[Title/Abstract] OR sensormedic[Title/Abstract] OR sensormedics[Title/Abstract] OR sensormedicus[Title/Abstract] OR sensormedix[Title/Abstract] OR sensorml[Title/Abstract] OR sensormotor[Title/Abstract] OR sensornet[Title/Abstract] OR sensornetworks[Title/Abstract] OR sensorneural[Title/Abstract] OR sensornodes[Title/Abstract] OR sensornye[Title/Abstract] OR sensoro[Title/Abstract] OR sensorograms[Title/Abstract] OR sensoromotor[Title/Abstract] OR sensoromotoric[Title/Abstract] OR sensoroneural[Title/Abstract] OR sensoror[Title/Abstract] OR sensorotoxin[Title/Abstract] OR sensorpoly[Title/Abstract] OR sensorproperties[Title/Abstract] OR sensorregulator[Title/Abstract] OR sensorreporters[Title/Abstract] OR sensorresponse[Title/Abstract] OR sensors[Title/Abstract] OR sensors'[Title/Abstract] OR sensors's[Title/Abstract] OR sensors2016[Title/Abstract] OR sensorsa[Title/Abstract] OR sensorsand[Title/Abstract] OR sensorsbased[Title/Abstract] OR sensorscope[Title/Abstract] OR sensorselection[Title/Abstract] OR sensorsetting[Title/Abstract] OR sensorship[Title/Abstract] OR sensorshowed[Title/Abstract] OR sensorsi[Title/Abstract] OR sensorsis[Title/Abstract] OR sensorspace[Title/Abstract] OR sensorsthe[Title/Abstract] OR sensorstrips[Title/Abstract] OR sensorsuse[Title/Abstract] OR sensorsv[Title/Abstract] OR sensorswere[Title/Abstract] OR sensort[Title/Abstract] OR sensortag[Title/Abstract] OR sensortalk[Title/Abstract] OR sensortech[Title/Abstract] OR sensortechnik[Title/Abstract] OR sensortechnology[Title/Abstract] OR sensortek[Title/Abstract] OR sensorthat[Title/Abstract] OR sensorthings[Title/Abstract] OR sensorto[Title/Abstract] OR sensortouch[Title/Abstract] OR sensortrade[Title/Abstract] OR sensorts[Title/Abstract] OR sensorwas[Title/Abstract] OR sensorweb[Title/Abstract] OR sensory[Title/Abstract] OR sensory'[Title/Abstract] OR sensory's[Title/Abstract] OR sensoryanalysis[Title/Abstract] OR sensorydeprivation[Title/Abstract] OR sensoryevoked[Title/Abstract] OR sensorygc[Title/Abstract] OR sensoryical[Title/Abstract] OR sensoryinteractions[Title/Abstract] OR sensorylike[Title/Abstract] OR sensorymodality[Title/Abstract] OR sensorymotor[Title/Abstract] OR sensorynav1[Title/Abstract] OR sensoryneural[Title/Abstract] OR sensoryneuropathy[Title/Abstract] OR sensoryomics[Title/Abstract] OR sensorypredominant[Title/Abstract] OR sensoryprocessing[Title/Abstract] OR sensoryrecovery[Title/Abstract] OR sensoryrelated[Title/Abstract] OR sensoryrhodopsin[Title/Abstract] OR sensoryrizotomy[Title/Abstract] OR sensorytransduction[Title/Abstract] OR sensorytreat[Title/Abstract] OR sensorytreat's[Title/Abstract])) OR (robot[Title/Abstract] OR robot'[Title/Abstract] OR robot'consists[Title/Abstract] OR robot'electronically[Title/Abstract] OR robot's[Title/Abstract] OR robota[Title/Abstract] OR robotac[Title/Abstract] OR robotanalyst[Title/Abstract] OR robotanalyst's[Title/Abstract] OR robotarium[Title/Abstract] OR robotassisted[Title/Abstract] OR robotassistedlaparoscopic[Title/Abstract] OR robotcar[Title/Abstract] OR robotechnologies[Title/Abstract] OR robotechnologist[Title/Abstract] OR roboteeg[Title/Abstract] OR roboter[Title/Abstract] OR roboterassistiert[Title/Abstract] OR roboterassistierte[Title/Abstract] OR roboterassistierter[Title/Abstract] OR robotfoto[Title/Abstract] OR robotham[Title/Abstract] OR robotherapist[Title/Abstract] OR robotherapist'[Title/Abstract] OR robotherapy[Title/Abstract] OR robothics[Title/Abstract] OR roboti[Title/Abstract] OR robotic[Title/Abstract] OR robotic'[Title/Abstract] OR robotica[Title/Abstract] OR robotical[Title/Abstract] OR robotically[Title/Abstract] OR roboticallyassisted[Title/Abstract] OR roboticamente[Title/Abstract] OR roboticapproach[Title/Abstract] OR roboticarm[Title/Abstract] OR roboticas[Title/Abstract] OR roboticassisted[Title/Abstract] OR roboticbed[Title/Abstract] OR roboticbird[Title/Abstract] OR roboticians[Title/Abstract] OR roboticist[Title/Abstract] OR roboticists[Title/Abstract] OR roboticists'[Title/Abstract] OR roboticization[Title/Abstract] OR roboticized[Title/Abstract] OR roboticizes[Title/Abstract] OR robotico[Title/Abstract] OR roboticos[Title/Abstract] OR roboticpancreatoduodenectomy[Title/Abstract] OR robotics[Title/Abstract] OR robotics'[Title/Abstract] OR roboticslab[Title/Abstract] OR roboticsurgery[Title/Abstract] OR roboticsystem[Title/Abstract] OR robotik[Title/Abstract] OR robotiker[Title/Abstract] OR robotiq[Title/Abstract] OR robotique[Title/Abstract] OR robotiquette[Title/Abstract] OR robotiquette'[Title/Abstract] OR robotisation[Title/Abstract] OR robotisches[Title/Abstract] OR robotise[Title/Abstract] OR robotised[Title/Abstract] OR robotisee[Title/Abstract] OR robotitian[Title/Abstract] OR robotix[Title/Abstract] OR robotization[Title/Abstract] OR robotize[Title/Abstract] OR robotized[Title/Abstract] OR robotizing[Title/Abstract] OR robotizzato[Title/Abstract] OR robotless[Title/Abstract] OR robotlike[Title/Abstract] OR robotmajsebeszet[Title/Abstract] OR robotmanipulators[Title/Abstract] OR robotmediated[Title/Abstract] OR robotnikinin[Title/Abstract] OR robotnon[Title/Abstract] OR roboto[Title/Abstract] OR robotocs[Title/Abstract] OR robotok[Title/Abstract] OR robotol[Title/Abstract] OR robototherapy[Title/Abstract] OR robotrac[Title/Abstract] OR robotrat[Title/Abstract] OR robotreviewer[Title/Abstract] OR robotreviewer's[Title/Abstract] OR robotripping[Title/Abstract] OR robotrobot[Title/Abstract] OR robotron[Title/Abstract] OR robots[Title/Abstract] OR robots'[Title/Abstract] OR robots'designs[Title/Abstract] OR robotsci[Title/Abstract] OR robotscientist[Title/Abstract] OR robotsebeszet[Title/Abstract] OR robotsebeszeti[Title/Abstract] OR robotsfor[Title/Abstract] OR robotti[Title/Abstract] OR robottom[Title/Abstract] OR robottrade[Title/Abstract] OR robotuna[Title/Abstract] OR robotutor[Title/Abstract] OR robotworld[Title/Abstract])) OR internet[Title/Abstract]) OR social media[Title/Abstract]) OR (phone[Title/Abstract] OR phone'[Title/Abstract] OR phone's[Title/Abstract] OR phonear[Title/Abstract] OR phonebased[Title/Abstract] OR phonebook[Title/Abstract] OR phoneburst[Title/Abstract] OR phonecall[Title/Abstract] OR phonecard[Title/Abstract] OR phonecardiographic[Title/Abstract] OR phonecians[Title/Abstract] OR phonecians'[Title/Abstract] OR phonectically[Title/Abstract] OR phoned[Title/Abstract] OR phonedependency[Title/Abstract] OR phoneeded[Title/Abstract] OR phoneeutria[Title/Abstract] OR phonefriend[Title/Abstract] OR phonegap[Title/Abstract] OR phoneigen[Title/Abstract] OR phonein[Title/Abstract] OR phoneix[Title/Abstract] OR phonelectrocardiography[Title/Abstract] OR phoneline[Title/Abstract] OR phonem[Title/Abstract] OR phonema[Title/Abstract] OR phonemas[Title/Abstract] OR phonematic[Title/Abstract] OR phonematically[Title/Abstract] OR phonematics[Title/Abstract] OR phonematique[Title/Abstract] OR phonemchanograms[Title/Abstract] OR phoneme[Title/Abstract] OR phoneme'[Title/Abstract] OR phoneme's[Title/Abstract] OR phonemegrapheme[Title/Abstract] OR phonemena[Title/Abstract] OR phonemes[Title/Abstract] OR phonemes'[Title/Abstract] OR phonemeter[Title/Abstract] OR phonemethod[Title/Abstract] OR phonemic[Title/Abstract] OR phonemicably[Title/Abstract] OR phonemically[Title/Abstract] OR phonemicisation[Title/Abstract] OR phonemicization[Title/Abstract] OR phonemics[Title/Abstract] OR phonemisation[Title/Abstract] OR phonemoformation[Title/Abstract] OR phonemonon[Title/Abstract] OR phonemotopic[Title/Abstract] OR phonems[Title/Abstract] OR phonendoscope[Title/Abstract] OR phonendoscopes[Title/Abstract] OR phonendoscopic[Title/Abstract] OR phonenix[Title/Abstract] OR phonenterographia[Title/Abstract] OR phoneomic[Title/Abstract] OR phonequant[Title/Abstract] OR phonequit[Title/Abstract] OR phonereporting[Title/Abstract] OR phonernically[Title/Abstract] OR phonerpeton[Title/Abstract] OR phones[Title/Abstract] OR phones'[Title/Abstract] OR phones4u[Title/Abstract] OR phoneshop[Title/Abstract] OR phoneside[Title/Abstract] OR phonesimulated[Title/Abstract] OR phonesis[Title/Abstract] OR phonesoap[Title/Abstract] OR phonesonhuman[Title/Abstract] OR phonesthemes[Title/Abstract] OR phonesthemic[Title/Abstract] OR phonet[Title/Abstract] OR phonethep[Title/Abstract] OR phonetic[Title/Abstract] OR phonetica[Title/Abstract] OR phonetical[Title/Abstract] OR phonetically[Title/Abstract] OR phoneticeffects[Title/Abstract] OR phonetician[Title/Abstract] OR phonetician's[Title/Abstract] OR phoneticians[Title/Abstract] OR phoneticization[Title/Abstract] OR phoneticized[Title/Abstract] OR phonetics[Title/Abstract] OR phonetics'[Title/Abstract] OR phonetik[Title/Abstract] OR phonetion[Title/Abstract] OR phonetique[Title/Abstract] OR phonetisation[Title/Abstract] OR phonetogram[Title/Abstract] OR phonetograms[Title/Abstract] OR phonetograph[Title/Abstract] OR phonetographic[Title/Abstract] OR phonetography[Title/Abstract] OR phonetometric[Title/Abstract] OR phonetoxin[Title/Abstract] OR phonetype[Title/Abstract] OR phonetypical[Title/Abstract] OR phoneus[Title/Abstract] OR phoneuse[Title/Abstract] OR phoneutria[Title/Abstract] OR phoneutriatoxin[Title/Abstract] OR phoneutrism[Title/Abstract] OR phoney[Title/Abstract] OR phoneyusa[Title/Abstract])) OR (telephone[Title/Abstract] OR telephone'[Title/Abstract] OR telephone's[Title/Abstract] OR telephoneassisted[Title/Abstract] OR telephonecare[Title/Abstract] OR telephoned[Title/Abstract] OR telephoneear[Title/Abstract] OR telephoneinterviews[Title/Abstract] OR telephoners[Title/Abstract] OR telephoners'[Title/Abstract] OR telephones[Title/Abstract] OR telephonesex[Title/Abstract] OR telephonetics[Title/Abstract])) OR online[Title/Abstract]) OR (ipad[Title/Abstract] OR ipad's[Title/Abstract] OR ipad120[Title/Abstract] OR ipad2[Title/Abstract] OR ipad20[Title/Abstract] OR ipad2s[Title/Abstract] OR ipad4[Title/Abstract] OR ipada[Title/Abstract] OR ipadair[Title/Abstract] OR ipadam[Title/Abstract] OR ipadar[Title/Abstract] OR ipadb[Title/Abstract] OR ipade[Title/Abstract] OR ipadech[Title/Abstract] OR ipadh[Title/Abstract] OR ipadia[Title/Abstract] OR ipadm[Title/Abstract] OR ipadnych[Title/Abstract] OR ipado[Title/Abstract] OR ipados[Title/Abstract] OR ipads[Title/Abstract] OR ipadstrade[Title/Abstract] OR ipadt[Title/Abstract] OR ipadtrade[Title/Abstract] OR ipadu[Title/Abstract] OR ipadur1[Title/Abstract] OR ipadvas[Title/Abstract])) OR (computer[Title/Abstract] OR computer'[Title/Abstract] OR computer's[Title/Abstract] OR computer18[Title/Abstract] OR computer19[Title/Abstract] OR computer3[Title/Abstract] OR computeradapted[Title/Abstract] OR computeraided[Title/Abstract] OR computerally[Title/Abstract] OR computeranalysis[Title/Abstract] OR computerand[Title/Abstract] OR computerangiography[Title/Abstract] OR computeranimated[Title/Abstract] OR computerarthrometry[Title/Abstract] OR computerassisted[Title/Abstract] OR computerassistierte[Title/Abstract] OR computerbase[Title/Abstract] OR computerbased[Title/Abstract] OR computercalculated[Title/Abstract] OR computerchemie[Title/Abstract] OR computerclassified[Title/Abstract] OR computercode[Title/Abstract] OR computercontrolled[Title/Abstract] OR computerd[Title/Abstract] OR computerdesigned[Title/Abstract] OR computerdocking[Title/Abstract] OR computerdokumentation[Title/Abstract] OR computerdriven[Title/Abstract] OR computered[Title/Abstract] OR computerenhanced[Title/Abstract] OR computerese[Title/Abstract] OR computereyes[Title/Abstract] OR computerezed[Title/Abstract] OR computerfiled[Title/Abstract] OR computerfitted[Title/Abstract] OR computergame[Title/Abstract] OR computergames[Title/Abstract] OR computergenerated[Title/Abstract] OR computergestutzte[Title/Abstract] OR computergestutzter[Title/Abstract] OR computergraphic[Title/Abstract] OR computergraphical[Title/Abstract] OR computergraphically[Title/Abstract] OR computergraphics[Title/Abstract] OR computergraphs[Title/Abstract] OR computerguided[Title/Abstract] OR computerheld[Title/Abstract] OR computeric[Title/Abstract] OR computerin[Title/Abstract] OR computering[Title/Abstract] OR computerintensive[Title/Abstract] OR computerinterpolated[Title/Abstract] OR computeris[Title/Abstract] OR computerisation[Title/Abstract] OR computerisations[Title/Abstract] OR computerise[Title/Abstract] OR computerised[Title/Abstract] OR computerised'[Title/Abstract] OR computerisedprovider[Title/Abstract] OR computerisee[Title/Abstract] OR computerises[Title/Abstract] OR computerising[Title/Abstract] OR computerism[Title/Abstract] OR computerizability[Title/Abstract] OR computerizable[Title/Abstract] OR computerizada[Title/Abstract] OR computerization[Title/Abstract] OR computerization'[Title/Abstract] OR computerization's[Title/Abstract] OR computerizd[Title/Abstract] OR computerize[Title/Abstract] OR computerizea[Title/Abstract] OR computerized[Title/Abstract] OR computerized'[Title/Abstract] OR computerizedcardiotocography[Title/Abstract] OR computerizeddynamic[Title/Abstract] OR computerizeds[Title/Abstract] OR computerizedt[Title/Abstract] OR computerizedtomographic[Title/Abstract] OR computerizes[Title/Abstract] OR computerizewd[Title/Abstract] OR computerizied[Title/Abstract] OR computerizing[Title/Abstract] OR computerizsed[Title/Abstract] OR computerizzata[Title/Abstract] OR computerlab[Title/Abstract] OR computerlabelled[Title/Abstract] OR computerland[Title/Abstract] OR computerless[Title/Abstract] OR computerlike[Title/Abstract] OR computerlink[Title/Abstract] OR computerlink's[Title/Abstract] OR computerly[Title/Abstract] OR computermediated[Title/Abstract] OR computermen[Title/Abstract] OR computermotion[Title/Abstract] OR computernavigated[Title/Abstract] OR computernavigation[Title/Abstract] OR computerneuron[Title/Abstract] OR computero[Title/Abstract] OR computerogenic[Title/Abstract] OR computerologist[Title/Abstract] OR computeromography[Title/Abstract] OR computeros[Title/Abstract] OR computerpart[Title/Abstract] OR computerphobe[Title/Abstract] OR computerphobia[Title/Abstract] OR computerphobia'[Title/Abstract] OR computerphobic[Title/Abstract] OR computerprogram[Title/Abstract] OR computerprogrammen[Title/Abstract] OR computerprogrammes[Title/Abstract] OR computerprograms[Title/Abstract] OR computerreadable[Title/Abstract] OR computerrelated[Title/Abstract] OR computerrhea[Title/Abstract] OR computerrised[Title/Abstract] OR computers[Title/Abstract] OR computers'[Title/Abstract] OR computers''[Title/Abstract] OR computerscan[Title/Abstract] OR computerscreen[Title/Abstract] OR computersimulation[Title/Abstract] OR computersimulations[Title/Abstract] OR computersmartphone[Title/Abstract] OR computersonography[Title/Abstract] OR computerspeak[Title/Abstract] OR computerspiel[Title/Abstract] OR computerspielabhangigkeit[Title/Abstract] OR computerspielsucht[Title/Abstract] OR computerstored[Title/Abstract] OR computersupported[Title/Abstract] OR computersystem[Title/Abstract] OR computersystems[Title/Abstract] OR computertime[Title/Abstract] OR computertized[Title/Abstract] OR computertomografie[Title/Abstract] OR computertomografischen[Title/Abstract] OR computertomogram[Title/Abstract] OR computertomogramm[Title/Abstract] OR computertomogramms[Title/Abstract] OR computertomograms[Title/Abstract] OR computertomograph[Title/Abstract] OR computertomographic[Title/Abstract] OR computertomographical[Title/Abstract] OR computertomographically[Title/Abstract] OR computertomographie[Title/Abstract] OR computertomographies[Title/Abstract] OR computertomographs[Title/Abstract] OR computertomography[Title/Abstract] OR computerunterstutzte[Title/Abstract] OR computerunterstutztes[Title/Abstract] OR computerusage[Title/Abstract] OR computerville[Title/Abstract] OR computervirus[Title/Abstract] OR computervision[Title/Abstract] OR computerworld[Title/Abstract])) OR (electronic[Title/Abstract] OR electronic'[Title/Abstract] OR electronic's[Title/Abstract] OR electronica[Title/Abstract] OR electronical[Title/Abstract] OR electronicall[Title/Abstract] OR electronically[Title/Abstract] OR electronically'[Title/Abstract] OR electronicallyexcited[Title/Abstract] OR electronicaly[Title/Abstract] OR electronicas[Title/Abstract] OR electronicbibliographic[Title/Abstract] OR electroniccards[Title/Abstract] OR electroniccase[Title/Abstract] OR electronicdatabase[Title/Abstract] OR electronicdocument[Title/Abstract] OR electronicfans[Title/Abstract] OR electroniclly[Title/Abstract] OR electronicmaterials[Title/Abstract] OR electronicmedical[Title/Abstract] OR electronicmedsman[Title/Abstract] OR electronicmessage[Title/Abstract] OR electronico[Title/Abstract] OR electronicos[Title/Abstract] OR electronicpatientrecords[Title/Abstract] OR electronicplatform[Title/Abstract] OR electronicquestionnaire[Title/Abstract] OR electronicreadout[Title/Abstract] OR electronicrecruitment[Title/Abstract] OR electronicrograph[Title/Abstract] OR electronics[Title/Abstract] OR electronics'[Title/Abstract] OR electronicsearch[Title/Abstract] OR electronicssensors[Title/Abstract] OR electronictemplate[Title/Abstract] OR electronicwaste[Title/Abstract] OR electronicwe[Title/Abstract])) OR web[Title/Abstract])) AND ((Clinical Trial[ptyp] OR Controlled Clinical Trial[ptyp] OR Journal Article[ptyp] OR Meta-Analysis[ptyp] OR Observational Study[ptyp] OR Randomized Controlled Trial[ptyp] OR Review[ptyp] OR systematic[sb]) AND ("2010/01/01"[PDAT] : "2019/07/31"[PDAT]) AND "humans"[MeSH Terms])

**EMBASE**

| 1. loneliness/ |
| --- |
| 2. Loneliness.ti,ab. |
| 3. (lonely or social isolation).ti,ab. |
| 4. 1 or 2 or 3 |
| 5. (digital* or technol* or sensor* or robot* or internet* or social media or smartphone* or smart phone* or telephone* or phone* or online or ipad* or computer* or electronic* or Web).ti,ab. |
| 6. robotics/ |
| 7. Internet/ |
| 8. social media/ |
| 9. smartphone/ |
| 10. personal digital assistant/ |
| 11. computer/ |
| 12. telephone/ |
| 13. 5 or 6 or 7 or 8 or 9 or 10 or 11 or 12 |
| 14. 4 and 13 |
| 15. limit 14 to (human and english language) |
| 16. limit 15 to yr="2010 -Current" |

**MEDLINE**

| 1. LONELINESS/ |
| --- |
| 2. Loneliness.ti,ab. |
| 3. (lonely or social isolation).ti,ab. |
| 4. 1 or 2 or 3 |
| 5. (digital* or technol* or sensor* or robot* or internet* or social media or smartphone* or smart phone* or telephone* or phone* or online or ipad* or computer* or electronic* or Web).ti,ab. |
| 6. ROBOTICS/ |
| 7. INTERNET/ |
| 8. Social Media/ |
| 9. TELEPHONE/ |
| 10. Smartphone/ |
| 11. Computers, Handheld/ |
| 12. COMPUTERS/ |
| 13. 5 or 6 or 7 or 8 or 9 or 10 or 11 or 12 |
| 14. 4 and 13 |
| 15. limit 14 to (english language and humans and yr="2010 -Current" and (clinical trial, all or evaluation studies or journal article or meta analysis or observational study or "review" or systematic reviews)) |

**CINAHL**

**Interface** - EBSCOhost Research Databases
**Search Screen** - Advanced Search
**Database** - CINAHL

((MH loneliness) OR (TX loneliness) OR ((TX lonely) OR (TX social isolation))) AND ((TX (digital* OR technol* OR sensor* OR robot* OR social media OR smartphone* OR smart phone* OR phone* OR online OR ipad* OR computer* OR elctronic* OR Web)) OR (MH technology) OR (MH robotics) OR (MH internet) OR (MH social media) OR (MH smartphone) OR (MH telephone) OR (MH computers, hand-held) OR (MH computers, portable) OR (MH world wide web))

**WEB OF SCIENCE**

| \|  \| \| --- \| | |
| --- | --- | --- |
| # 1 | **TOPIC:** (loneliness) *OR* **TOPIC:** (lonely) *OR* **TOPIC:** (social isolation) |
|  | *Indexes=SCI-EXPANDED, SSCI, A&HCI, ESCI Timespan=1900-2019* |
| # 2 | **TOPIC:** (digital) *OR* **TOPIC:** (technolog*) *OR* **TOPIC:** (sensor*) *OR* **TOPIC:** (robot*) *OR* **TOPIC:** (internet) *OR* **TOPIC:** (social media) *OR* **TOPIC:** (smart phone*) *OR* **TOPIC:** (online) *OR* **TOPIC:** (ipad*) *OR* **TOPIC:** (computer*) *OR* **TOPIC:** (electronic*) *OR* **TOPIC:** (web) |
|  | *Indexes=SCI-EXPANDED, SSCI, A&HCI, ESCI Timespan=1900-2019* |
| # 3 | #2  AND #1 |
|  | *Indexes=SCI-EXPANDED, SSCI, A&HCI, ESCI Timespan=1900-2019* |
| # 4 | #2  AND #1 |
|  | **Refined by:** **PUBLICATION YEARS:** ( 2019 OR 2018 OR 2017 OR 2016 OR 2015 OR 2014 OR 2013 OR 2012 OR 2011 OR 2010 ) |
|  | *Indexes=SCI-EXPANDED, SSCI, A&HCI, ESCI Timespan=1900-2019* |
| # 5 | #2  AND #1 |
|  | **Refined by:** **PUBLICATION YEARS:** ( 2019 OR 2018 OR 2017 OR 2016 OR 2015 OR 2014 OR 2013 OR 2012 OR 2011 OR 2010 ) AND **DOCUMENT TYPES:** ( ARTICLE OR REVIEW ) |
|  | *Indexes=SCI-EXPANDED, SSCI, A&HCI, ESCI Timespan=1900-2019* |
| # 6 | #2  AND #1 |
|  | **Refined by:** **PUBLICATION YEARS:** ( 2019 OR 2018 OR 2017 OR 2016 OR 2015 OR 2014 OR 2013 OR 2012 OR 2011 OR 2010 ) AND **DOCUMENT TYPES:** ( ARTICLE OR REVIEW ) AND **LANGUAGES:** ( ENGLISH ) |
|  | *Indexes=SCI-EXPANDED, SSCI, A&HCI, ESCI Timespan=1900-2019* |

**Database:** Web of Science Core Collection.
